# Supplementary material for: Complexome profiling of the Chlamydomonas psb28 mutant reveals TEF5 as an early PSII assembly factor
Source: Plant Cell. 2025 Mar 17;37(6):koaf055. doi: 10.1093/plcell/koaf055 (PMC12164590; doi:10.1093/plcell/koaf055)
Supplement: koaf055_Supplementary_Data [file koaf055_supplementary_data.zip › psb28_interactive.zip!/plots/intensity/qualityPlots/peptides/SEBP1_Cre03.g185550.t1.2.html]

### Cre03.g185550.t1.2

Trivial name: SEBP1  
SeqCoverage [median]: 9.10  
Mass: 41.45 kDa  
  
 

| Sequence | MissedCleavage | Mass [Da] | wtIntensity | muIntensity | Score |
| --- | --- | --- | --- | --- | --- |
| ATFDNPAYER | 0 | 1182.53 | 0.00 | 1296.37 | 56.73 |
| EQVAAGMGIYGPR | 0 | 1347.66 | 0.00 | 714.63 | 108.67 |
| GVFTNVTSPTTK | 0 | 1250.65 | 0.00 | 449.11 | 107.21 |
|  |  |  | 0.0 | 2460.1 |  |
